# Activity of mefloquine and mefloquine derivatives against *Echinococcus multilocularis*

**DOI:** 10.1016/j.ijpddr.2018.06.004

**Published:** 2018-06-15

**Authors:** Reto Rufener, Dominic Ritler, Jana Zielinski, Luca Dick, Emerson Teixeira da Silva, Adriele da Silva Araujo, Deborah Elisabeth Joekel, David Czock, Christine Goepfert, Adriana Marques Moraes, Marcus Vinicius Nora de Souza, Joachim Müller, Meike Mevissen, Andrew Hemphill, Britta Lundström-Stadelmann

**Affiliations:** a Institute of Parasitology, Department of Infectious Diseases and Pathobiology, Vetsuisse Faculty, University of Bern, Länggassstrasse 122, 3012, Bern, Switzerland; b Division of Pharmacology and Toxicology, Vetsuisse Faculty, University of Bern, Länggassstrasse 124, 3012, Bern, Switzerland; c Fundação Oswaldo Cruz, Instituto de Tecnologia em Fármacos – Far Manguinhos, 21041-250, Rio de Janeiro, Brazil; d Institute of Parasitology, Vetsuisse Faculty, University of Zurich, Winterthurerstrasse 266a, 8057 Zurich, Switzerland; e Department of Clinical Pharmacology and Pharmacoepidemiology, University of Heidelberg, Im Neuenheimer Feld 410, 69120 Heidelberg, Germany; f Institute of Animal Pathology COMPATH, Department of Infectious Diseases and Pathobiology, Vetsuisse Faculty, University of Bern, Länggassstrasse 122, Switzerland

**Keywords:** Alveolar echinococcosis, Treatment, Anti-malaria, HPLC, Drug repurposing, Structure activity relationship

## Abstract

The cestode *E. multilocularis* causes the disease alveolar echinococcosis (AE) in humans. The continuously proliferating metacestode (larval stage) of the parasite infects mostly the liver and exhibits tumor-like growth. Current chemotherapeutical treatment options rely on benzimidazoles, which are rarely curative and have to be applied daily and life-long. This can result in considerable hepatotoxicity and thus treatment discontinuation. Therefore, novel drugs against AE are urgently needed. The anti-malarial mefloquine was previously shown to be active against *E. multilocularis* metacestodes *in vitro,* and in mice infected by intraperitoneal inoculation of metacestodes when administered at 100 mg/kg by oral gavage twice a week for 12 weeks. In the present study, the same dosage regime was applied in mice infected via oral uptake of eggs representing the natural route of infection. After 12 weeks of treatment, the presence of parasite lesions was assessed in a liver squeeze chamber and by PCR, and a significantly reduced parasite load was found in mefloquine-treated animals. Assessment of mefloquine plasma concentrations by HPLC and modeling using a two-compartment pharmacokinetic model with first-order absorption showed that >90% of the expected steady-state levels (C_min_ 1.15 mg/L, C_max_ 2.63 mg/L) were reached. These levels are close to concentrations achieved in humans during long-term weekly dosage of 250 mg (dose applied for malaria prophylaxis). *In vitro* structure-activity relationship analysis of mefloquine and ten derivatives revealed that none of the derivatives exhibited stronger activities than mefloquine. Activity was only observed, when the 2-piperidylmethanol group of mefloquine was replaced by an amino group-containing residue and when the trifluoromethyl residue on position 8 of the quinoline structure was present. This is in line with the anti-malarial activity of mefloquine and it implies that the mode of action in *E. multilocularis* might be similar to the one against malaria.

## Highlights

• Tested anti-malarial mefloquine against mice infected orally with *E. multilocularis*.
• Reduced liver lesion numbers upon bi-weekly treatment with 100 mg/kg mefloquine.
• Similar mefloquine levels reached in mice as in human malaria prophylaxis.
• Efficacy of mefloquine is dependent on two molecular residues.

## Graphical abstract

**Figure:**
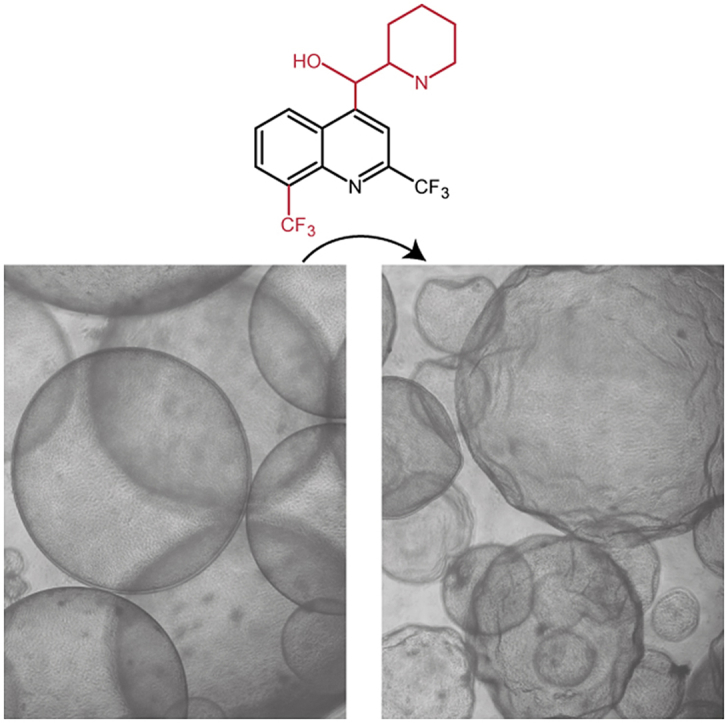

## Abbreviations

ABZ: albendazole
AE: alveolar echinococcosis
PGI: phosphoglucose isomerase
SH: sodium-hypochlorite
TLC: thin-layer chromatography

## 1 Introduction

The parasitic cestode *Echinococcus multilocularis* causes alveolar echinococcosis (AE) in humans and a variety of mammals, such as dogs, captive monkeys, beavers, and others (Deplazes and Eckert, 2001). *E. multilocularis* is found on the Northern hemisphere, including high endemicity areas in Central and Eastern Asia (e.g. Kyrgyzstan, China, and Northern Japan) as well as in Central and Eastern Europe (Deplazes et al., 2017). The total global burden of human AE was estimated to be 18′235 new cases per year (Torgerson et al., 2010). Over the recent decades, the parasite became more prevalent in Europe (Thompson et al., 2017) and Canada (Trotz-Williams et al., 2017). Especially in endemic areas with low standard health care systems, the disease poses an increasing and uncontrolled health problem (Kern et al., 2017).

Definitive hosts (mainly foxes, dogs, and raccoon dogs) harbor the adult stage of *E. multilocularis* in their intestines and this leads to contamination of the environment with infectious eggs. Intermediate hosts such as small rodents, but also humans (dead-end hosts), and other mammals, may accidentally acquire eggs containing an infectious oncosphere orally, and be infected with the parasite. Following infection, the oncosphere differentiates into the metacestode stage, primarily in the liver, where it infiltrates the adjacent tissue by asexual proliferation of vesicles. Metacestodes exhibit an unlimited reproductive potential, gradually forming cancer-like lumps, often with necrotic areas in the centre. Thus, human AE is a chronic disease with extensive morbidity and mortality if untreated (Kern et al., 2017). The only curative treatment for AE is complete surgical resection of the parasite tissue. Such invasive surgery is performed in about 30% of all AE patients, therefore most receive only continuous medication with the benzimidazole-derivatives albendazole (ABZ) or mebendazole (Kern et al., 2017). Benzimidazoles have drastically improved the life expectancy and quality of life of patients. Whereas the 10-years survival rate of untreated AE patients was 0–25% in the pre-benzimidazole era, benzimidazole-treated patients to date have a 10-years survival rate of 91–97% in countries with well-developed health-care (Ammann and Eckert, 1996; Grüner et al., 2017). However, benzimidazoles are mainly parasitostatic, requiring life-long administration to avoid recurrence. Benzimidazoles bind to beta tubulin and interfere in microtubule formation, thereby impairing uptake of nutrients and parasite growth (Lacey, 1990). However, stem cells in the germinal layer of *E. multilocularis* metacestodes express a beta tubulin isoform, TUB-2, which does not bind to benzimidazoles rendering stem cells largely resistant to the dosages of benzimidazoles used in standard treatments. This, in combination with the limited uptake and half-life of the drug, could, at least partially, explain the parasitostatic rather than parasiticidal action of benzimidazoles (Brehm and Koziol, 2014). A drawback of benzimidazole-based therapy is that about 16% of the treated patients experience adverse effects such as hepatotoxicity that lead to treatment-discontinuation (Steiger et al., 1990). ABZ treatment increases the host immune response against the parasite, implying that the action of benzimidazoles could also be dependent on the immune system (Ricken et al., 2017). With increasing numbers of patients and no alternative to benzimidazoles, the development of better and/or alternative treatment options becomes increasingly urgent (Kern et al., 2017). Two drugs that were studied in clinical trials against AE over the last years are the anti-fungal agent amphotericin B and the broad-spectrum anti-parasitic nitazoxanide, but they were not further pursued due to minimal activity in humans and pronounced side-effects (Kern et al., 2008; Tappe et al., 2009).

Pharmaceutical companies are reluctant to engage in preclinical drug development for AE, and therefore an important focus is on repurposing of existing drugs or compound classes that are on the market or being developed for other indications. This approach could result in lower costs, lower risk of failure, and faster time to the market within the drug development process (Andrews et al., 2014; Panic et al., 2014). A rich source for drug repurposing against parasites are anti-malarials, since over 6 million compounds have been screened for activity against the blood stage of *P. falciparum*, and over 20′000 hits with activity in the low μM range have been identified. Over the past years, several anti-malarials were shown to exhibit activity against *E. multilocularis* metacestodes (Lubinsky, 1969; Reuter et al., 2006; Spicher et al., 2008; Küster et al., 2014; Stadelmann et al., 2016), including mefloquine (Küster et al., 2011, 2015; Stadelmann et al., 2011). In *Plasmodium,* mefloquine inhibits the formation of hemozoin, an essential step in heme detoxification upon hemoglobin degradation (Egan et al., 1994). Additional proposed targets are the ribosomes (Wong et al., 2017), phosphatidylinositol, volume-regulated anion channels and endocytosis (Dassonville-Klimpt et al., 2011). Mefloquine is also active against the helminth parasite *Schistosoma* (Keiser and Utzinger, 2012), where inhibition of hemozoin formation (Corrêa Soares et al., 2009) as well as impairment of enolase activity (Manneck et al., 2012) were postulated as potential mechanisms of action. In addition, mefloquine is active against cancer cells (Sharma et al., 2012; Liu et al., 2016), and neuronal cells (Lim and Go, 1985; Cruikshank et al., 2004; McArdle et al., 2005; Milatovic et al., 2011). The adverse-effects of mefloquine are well-known. Mefloquine has been reported to induce a post-hepatic syndrome (including gastrointestinal disturbances, headache, malaise) (Mawson, 2013) and may induce neuropsychiatric side-effects in patients, who are either receiving malaria prophylaxis or single dose treatment (Croft and Herxheimer, 2002; Croft, 2007; Grabias and Kumar, 2016; Nevin and Byrd, 2016).

Upon *in vitro* treatment of *E. multilocularis* metacestodes with mefloquine, a rapid separation of the cellular germinal layer from the acellular laminated layer and collapse of the metacestode tissue was observed (Küster et al., 2011). Subsequent injection of *in vitro*-treated parasites into animals showed that the drug exhibited parasiticidal activity (Küster et al., 2011). To reduce the expected neurological side-effects *in vivo*, erythro-enantiomers of mefloquine were tested *in vitro*, as it was suggested that adverse effects might be attributable mainly to one form of enantiomer. However, against *E. multilocularis*, both enantiomers exhibited similar activities (Stadelmann et al., 2011). In the secondary infection model mice are infected by intraperitoneal inoculation of metacestodes, reflecting the late chronic, disseminated stage of disease. In this model we showed that intraperitoneal injection of mefloquine at 25 mg/kg twice per week during 8 weeks resulted in a significant reduction of the parasite burden (Küster et al., 2011). The same was achieved upon oral application of mefloquine at 100 mg/kg, twice per week for 12 weeks (Küster et al., 2015). Thus, mefloquine was active in the chronic disease model. However, there is no information on the drug plasma concentrations required for activity against murine AE in the above-mentioned studies. Further, mefloquine has not yet been assessed in a primary infection model, i.e. in mice infected orally with *E. multilocularis* eggs reflecting the natural route of infection and earlier stage of disease. *E. multilocularis* ferritin and cystatin were identified to possibly interact with mefloquine (Küster et al., 2015). However, this has not been further investigated and additional information on the mode of action of mefloquine against *E. multilocularis* is lacking to date.

We here assessed the anti-parasitic effect of mefloquine in a primary mouse infection model of AE, and measured drug plasma concentrations by high-performance liquid chromatography (HPLC). In addition, ten derivatives of the molecule were tested *in vitro* against *E. multilocularis* metacestodes to further investigate the mode of action and structure activity relationship of mefloquine.

## 2 Materials and methods

### 2.1 Materials

All chemicals were purchased from Sigma-Aldrich (Buchs, Switzerland), if not stated otherwise.

### 2.2 Isolation of *E.multilocularis* eggs and viability assessment

*E. multilocularis* eggs were obtained from naturally infected foxes during the regular Swiss hunting season in spring 2017 according to Hofer et al. (2000). In brief, adult *E. multilocularis* worms from the small intestines of foxes were collected and kept in 0.9% NaCl, the worms were squashed and the suspension was first passed through a 105 μm and a 41 μm diameter mesh, followed by a 21 μm mesh (Lanz-Anliker AG, Switzerland). The suspension was stored at 4 °C in PBS with 100 U penicillin and 100 μg streptomycin (Life Technologies, Switzerland) (PBS-P/S). The egg suspension was centrifuged every second to third week (500×*g*, 10 min, 4 °C), the supernatant was removed and replaced by fresh PBS-P/S. Integrity (maturity) of *Echinococcus* eggs was assessed by sodium hypochlorite resistance test (Deplazes and Eckert, 1988). In brief, 0.3 mL of a sodium-hypochlorite solution (2% active chlorine, pH 12) was added to 0.4 mL E. *multilocularis* egg suspension (500–1000 eggs/mL). The total number of eggs was determined in a McMaster-chamber. Few minutes after the addition of sodium-hypochlorite solution, oncospheres with intact membranes were counted. Sodium hypochlorite resistance was calculated from triplicate counts as percentage of intact oncospheres.

### 2.3 Animal housing and experimental infection with *E. multilocularis* eggs

All manipulations with animals followed the guidelines of the Swiss legislation on experimental animal procedures and the experiment was approved by the Bernese cantonal authorities under the license number BE112/14. Eight-week old female BALB/c mice (Charles River, Sulzfeld, Germany) weighing 20.4 ± 0.8 g at the beginning of the experiment were housed in temperature- and humidity-controlled animal facilities (biosafety level 2) with day/night cycle (12/12 h) and free access to water and food. Prior to egg infection, 35 mice were transferred to a biosafety level 3 animal facility and were infected by oral gavage of approximately 200 *E.* *multilocularis* eggs (corresponding to 46 viable eggs) suspended in 100 μL PBS. An additional 9 female BALB/c mice received oral gavage of 100 μL PBS only. After two weeks, animals were transferred back to a conventional biosafety level 2 facility.

### 2.4 Mefloquine treatment

The infected mice were randomly allocated into three egg-infected groups: (I) mefloquine treatment (n = 9); (II) albendazole (ABZ) treatment (n = 8); (III) placebo treatment (n = 9). Group (IV) consisted of the non-infected control group (n = 9). Based on power analysis in G*Power (version 3.1.9.2), a power of 0.8 and a p-value of 0.05, the minimal group size was calculated to n = 8. In groups I, II, and IV, we increased this number to n = 9, as for these groups plasma concentration assessments in three times three animals were planned (see section 2.7). At 4 weeks post infection (p.i.) mice were treated for a period of 12 weeks by oral gavage with drugs suspended in 100 μL corn oil. Mefloquine (Selleckchem, LuBioScience, Luzern, Switzerland) was applied at 100 mg/kg twice a week, and ABZ at 200 mg/kg during 5 consecutive days per week. The treatment schedule was as follows: mice in group (I) received mefloquine on day 1 and day 4, and corn oil without mefloquine on days 2, 3 and 5 of each week; to group (II) ABZ was applied on days 1–5 each week; group (III) received corn oil on days 1–5 each week; the uninfected mice in group (IV) were treated with mefloquine on day 1 and day 4, and with corn oil only on days 2, 3, and 5 of each week (as in group (I)), in order to evaluate mefloquine pharmacokinetics in uninfected mice. No treatments were performed on days 6 and 7 of each week. After 12 weeks of treatment, all animals were euthanized by CO_2_, livers were resected and cut into single liver lobes. Each liver lobe was placed into a squeeze chamber and presence of lesions was assessed in a blinded way using a stereo microscope. Lesion numbers of the three infected groups were analyzed by one-sided exact Wilcoxon rank-sum test using the R package coin version 1.2.2 (Hothorn et al., 2006) and p-values were Bonferroni adjusted (R version 3.4.2). The significance level was set to p < 0.05. Figures were prepared in Microsoft Excel (2010) and Adobe Illustrator 2015.1.0.

### 2.5 Histopathology

Histopathological analysis of liver tissues was performed from each mouse. Samples of the left lateral liver lobe were fixed for 24 h in 4% paraformaldehyde and paraffin embedded. Blocks were sectioned and stained with hematoxylin and eosin. Morphological changes on each section in relation to the controls were recorded. The microscopical evaluation was performed in a blinded fashion by a board-certified veterinary pathologist.

### 2.6 *E. multilocularis*-specific PCR of mouse livers

The presence or absence of *E. multilocularis* DNA in livers of infected mice was assessed by PCR. Each liver was cut into two pieces of similar size that were then treated equally. DNA was extracted using a commercial kit (NucleoSpin DNA RapidLyse; Macherey-Nagel, Oensingen, Switzerland). The samples were digested in 720 μL lysis buffer and 30 μL Proteinase K solution for 2 h at 65 °C. One glass bead of 5 mm in diameter was added prior to digestion and the samples were homogenized just before the start of the digestion and after 1 h of digestion in a FastPrep 24 Tissue lyser (MP Biomedicals, Eschwege, Germany) at 4 m/s for 60 s. DNA extraction was then continued according to the manufacturer's protocol with 160 μL of the digested samples. The extracted DNA was subsequently quantified in triplicates using the QuantiFluor dsDNA System (Promega, Dübendorf, Switzerland) according to the manufacturer's manual. Polymerase chain reaction (PCR) was performed according to Trachsel et al. (2007) with slight modifications applying the primers Cest1 and Cest2 (Eurofins Genomics, Ebersberg, Germany) of said study to amplify the mitochondrial NADH dehydrogenase subunit 1 gene. The amplification was done in a final volume of 20 μL reaction mixture (all components except the primers and samples were purchased from Promega), containing GoTaq Reaction buffer, 10 mM nucleotide mix, 1 U GoTaq G2 DNA Polymerase, 0.5 μM Cest1 primer, 0.5 μM Cest2 primer, and 1 μL sample. The PCR reactions were performed in a T3000 Thermocycler (Biometra, Göttingen, Germany) and had an initial denaturation at 94 °C for 3 min, followed by 35 cycles of denaturation at 94 °C for 30 s, annealing at 58 °C for 60 s, and elongation at 72 °C for 60 s, and a final elongation at 72 °C for 5 min. The PCR products were subsequently visualized in a 2% agarose gel with 0.2 μg/mL Ethidium bromide (Promega) under an UV illuminator.

### 2.7 Blood-sampling and sample extraction

At least 60 μL blood-samples were taken during the 12-week course of treatment from the tail vein of mice for subsequent analysis of mefloquine plasma concentrations. Blood samples were taken 1 and 5 weeks after treatment initiation (after doses 3 and 11 respectively). 12-week blood samples were taken by heart puncture (after dose 23) after euthanasia. At each of these time points, blood samples were retrieved 6, 24, and 48 h after mefloquine dosage from 3 mice in each group. Blood was taken with heparin-coated microvette tubes and plasma was retrieved by centrifugation for 15 min, 10′000×*g* at 4 °C. Each plasma sample was then spiked with the internal standard quinine (0.1 g/L in methanol) to 31.25 mg/L. All samples were immediately frozen on dry ice and stored at - 80 °C until analysis by HPLC. At every time point, an internal standard sample with quinine only was frozen in order to follow stability of the standard over time.

Plasma extraction and determination of mefloquine concentrations in mouse plasma were largely performed according to Ingram et al. (2013). For extraction, 1 mL acetonitrile was added to each plasma sample, and after short vortexing, samples were centrifuged at room temperature for 10 min at 10′000×*g*. Supernatants were collected and were dried for 2.5 h at 30 °C in an Eppendorf Concentrator 5301. Dried samples were reconstituted in 110 μL acetonitrile/potassium dihydrogen phosphate buffer (1:1 mix, potassium dihydrogen phosphate buffer 0.05 M, pH 3.9, pH adjusted with 0.05% phosphoric acid). After centrifugation (13′000×*g*, 10 min at room temperature), samples were transferred into conical HPLC cuvettes (0.2 mL, 6 × 31 mm, wide opening, Macherey Nagel) and were immediately subjected to HPLC.

### 2.8 Mefloquine standard curve for HPLC

A calibration curve was established by spiking of plasma from non-treated mice with a 1:2 dilution series of mefloquine from 25 to 0.098 mg/L (stock in acetonitrile/potassium dihydrogen phosphate), covering the range of the plasma samples. All calibration samples included the internal quinine standard as described above. Three standard curves were prepared independently. All standard samples were extracted exactly as stated for the plasma samples above.

### 2.9 Mefloquine plasma concentration measurements by HPLC

HPLC was performed as described previously with adaptations (Ingram et al., 2013). Mefloquine concentrations were analyzed on an Ultimate 3000 System (Dionex, Reinach, Switzerland) with an EC 250/4 Nucleodur 100-5 C18ec (Macherey Nagel) and UV detection at 284 nm. The mobile phase consisted of 35% methanol, 25% acetonitrile, and 40% potassium dihydrogen phosphate (pH 3.9). Column temperature was 25 °C, flow rate constant at 1 mL/min and each run was 10 min. The recorded peaks were annotated according to the retention times of known standards. Stability of samples was assessed with the help of the internal quinine standard. Mefloquine plasma concentrations were quantified based on internal calibration of the peak area to the internal standard quinine and calculated with a linear calibration curve in the software Chromeleon Ultimate 3000 (Dionex, CA, USA) and Microsoft Excel 2010. Further calculations and figures were prepared in SigmaPlot Version 14, and in Adobe Illustrator 2015.1.0.

### 2.10 Pharmacokinetic model

Mefloquine concentrations were modeled using a standard two-compartment pharmacokinetic model with first-order absorption. Mean mefloquine concentrations and a mean dose of 2.04 mg were used for calculations. Primary parameters were the absorption rate constant k_a_, the apparent clearance after extravascular administration CL/F, the intercompartment clearance CL_d_/F, and the apparent volumes of the central and peripheral compartment V_1_/F and V_2_/F. A secondary parameter was the terminal elimination half-life T_½_. Expected steady-state minimum (C_min_) and maximum (C_max_) concentrations were derived by simulating continued mefloquine dosing. Pharmacokinetic calculations were done using Phoenix WinNonlin 7.0 (Certara, Princeton, NJ, USA) and Figures prepared in Microsoft Excel (2010) and in Adobe Illustrator 2015.1.0.

### 2.11 Synthesis of mefloquine derivatives

Melting points were determined with a MQAPF-302 Micro Química apparatus and are uncorrected. NMR spectra were determined using 400 or 500 MHz Bruker AC spectrometers using tetramethylsylane as internal standard. Splitting patterns are as follows: s, singlet; d, duplet; t, triplet; quin, quintet; m, multiplet; Brl, broad signal. Infrared spectra were obtained using a Thermo Nicolet 6700 spectrometer. Mass spectra were recorded on Agilent 122 5532 GC/MS column by electron impact and high resolution spectra on a Bruker compact-TOF. The progress of the reactions was monitored by thin-layer chromatography (TLC) on 2.0 × 6.0 cm aluminum sheets (silica gel 60, HF-254, Merck) with a thickness of 0.25 mm, ultraviolet light irradiation. For column chromatography, a Merck silica gel (70–230 mesh) was used. Solvents and reagents were used without further purification.

**Fig. 1:**
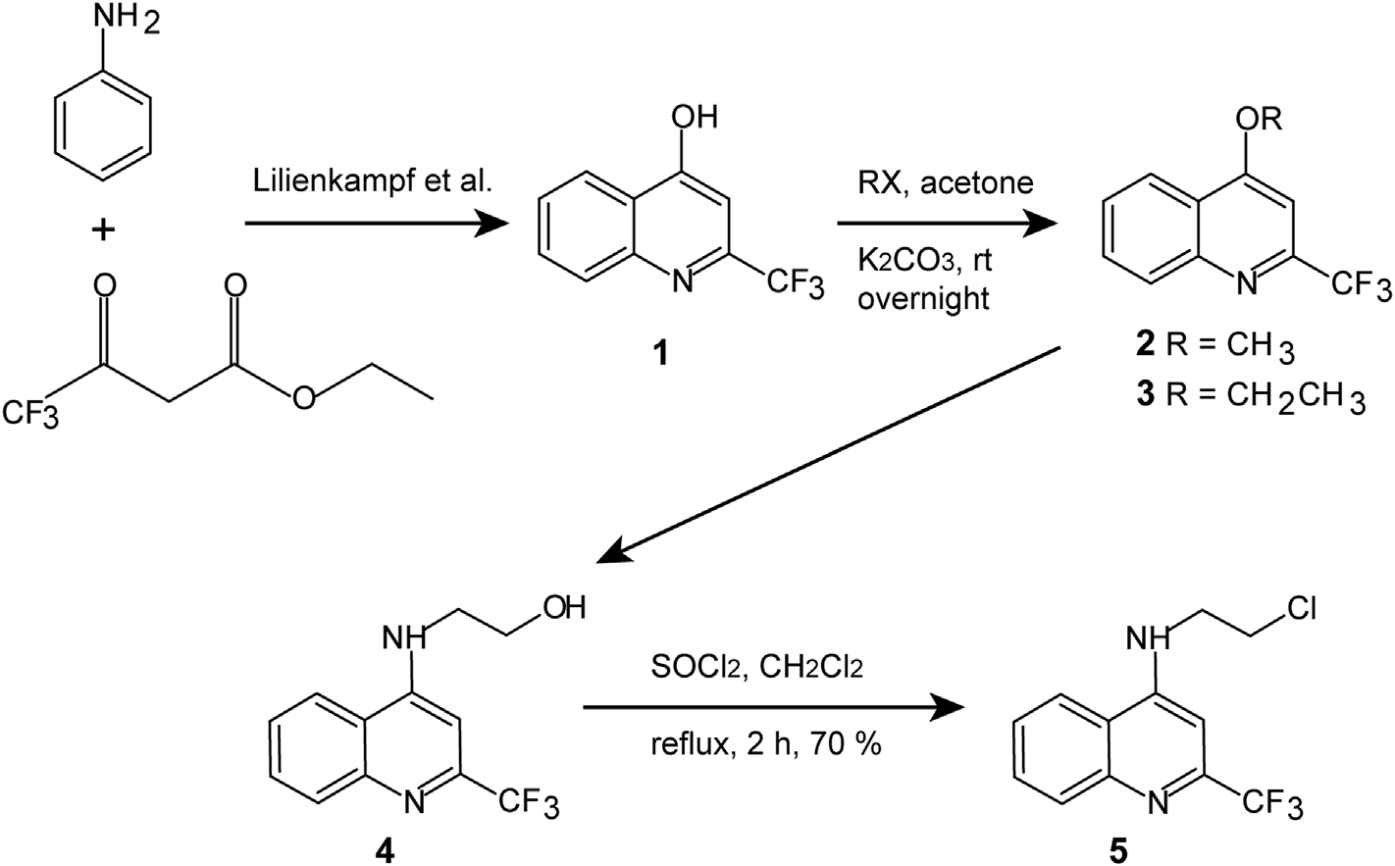
Synthesis of mefloquine derivatives based on the C-4 position.

10 derivatives of mefloquine were synthesized to be compared to mefloquine *in vitro* activity against *E. multilocularis*. The synthesis of six of them (PASALR-01-095, PASALR-01-097, MEFLOMETIL-02, PASALR-01-146, PASALR-01-096, PASALR-01-126) has been described elsewhere (see Table 1) (Barbosa-Lima et al., 2017; Lilienkampf et al., 2009). The other four derivatives were synthesized as described below and given in scheme in Fig. 1.

**Table 1:** List of ten mefloquine derivatives and synthesis.


abbreviation	full name	R^1^	R^2^	source
Mefloquine	(2,8-bis(trifluoromethyl)quinolin-4-yl)-piperidin-2-yl-methanol	-CF_3_	-CHOHPip	Selleckchem
PASALR-01-095	ethyl 2-((2,8-bis(trifluoromethyl)quinolin-4-yl)oxy)acetate	-CF_3_	-OCH_2_CO_2_Et	Acros Organics, according to (Lilienkampf et al., 2009)
PAMMLR-01-99.2	4-methoxy-2-(trifluoromethyl)quinoline	-H	-OCH_3_	**2**, Fig. 1
PASALR-01-097	*N*1-(2,8-bis(trifluoromethyl)quinolin-4-yl)ethane-1,2-diamine	-CF_3_	-NHCH_2_CH_2_NH_2_	(Barbosa-Lima et al., 2017)
MEFLOMETIL-02	4-methoxy-2,8-bis(trifluoromethyl)quinoline	-CF_3_	-OCH_3_	(Barbosa-Lima et al., 2017)
PASALR-01-137	4-ethoxy-2-(trifluoromethyl)quinoline	-H	-OEt	**3**, Fig. 1
PASALR-01-146	*N*-(2-chloroethyl)-2,8-bis(trifluoromethyl)quinolin-4-amine	-CF_3_	-NHCH_2_CH_2_Cl	(Barbosa-Lima et al., 2017)
PAMMLR-01-102-2	2-((2-(trifluoromethyl)quinolin-4-yl)amino)ethanol	-H	-NHCH_2_CH_2_OH	**4**, Fig. 1
PASALR-01-144	*N*-(2-chloroethyl)-2-(trifluoromethyl)quinolin-4-amine	-H	-NHCH_2_CH_2_Cl	**5**, Fig. 1
PASALR-01-096	*N*-butyl-2,8-bis(trifluoromethyl)quinolin-4-amine	-CF_3_	-NHbutyl	(Barbosa-Lima et al., 2017)
PASALR-01-126	2-((2,8-bis(trifluoromethyl)quinolin-4-yl)amino)ethanol	-CF_3_	-NHCH_2_CH_2_OH	(Barbosa-Lima et al., 2017)

#### 2.11.1 2-(trifluoromethyl)quinolin-4-ol 1

Polyphosphoric acid (11.25 g; 5 w/w) was added to an equimolar solution of aniline (2.25 g; 24.19 mmol) and ethyl 4,4,4-trifluoroacetoacetate (3.0 g; 24.19 mmol). The reaction mixture was stirred at 150 °C for 2 h. Reaction completion was monitored by TLC. The reaction mixture was slowly poured into ice water (500 mL) with vigorous stirring and stirred at room temperature during 30 min. The precipitated solid was filtered, washed with 50 mL water and dried in a vacuum oven for 4 h to get the crude product as white solid. The crude product (termed phenol **1**) was taken as such for the next step without further purification. Melting point (m.p.) 205–207 °C (lit.: 208–210 °C). Phenol **1** was not tested against *E. multilocularis*, but was needed for further synthesis.

#### 2.11.2 4-Methoxy-2-(trifluoromethyl)quinoline (PAMMLR-01-99.2)

The phenol **1** (3.0 g; 14.08 mmol) was dissolved in acetone (70 mL) by stirring at room temperature. K_2_CO_3_ (6 eq. (11.66) g, 84.48 mmol) and methyl iodide (5 eq., 4.3 mL, 70.4 mmol) were then added. The reaction was stirred overnight at room temperature. Then, the solvent was removed under reduced pressure and 70 mL water was added. The aqueous phase was extracted with ethyl acetate (3 × 60 mL) and the combined organic phases were dried over anhydrous Na_2_SO_4_ and concentrated under reduced pressure to leave a solid. This was purified by flash chromatography (SiO_2_, 230–400 mesh, AcOEt/nHex 5–10%) to furnish the compound 4-methoxy-2-(trifluoromethyl)quinoline (compound **2** in Fig. 1) as a pale white solid in 70% yield. m. p.: 99–103 °C. ^1^H NMR (MeOD, 500 MHz)δ: 8.27 (1H, d, *J* = 8.5 Hz), 8.08 (1H, d, *J* = 8.5 Hz), 7.85 (1H, t, *J* = 7.3 Hz), 7.67 (1H, t, *J* = 7.3 Hz), 7.28 (1H, s), 4.17 (3H, s, -OCH_3_). ^13^C NMR (MeOD, 125 MHz) d: 164.47, 148.83 (CF_3_, *J* = 34 Hz), 147.72, 131.95, 131.00, 128.42, 127.49, 121.63, 121.54, 96.08, 55.83. (IR *v* cm^−1^: 1315 (C-O-C). Theoretical mass calculated for [C11H8F3NO + H]: 228.0636; Found: 228.0634.

#### 2.11.3 4-Ethoxy-2-(trifluoromethyl)quinoline (PASALR-01-137)

The phenol **1** (3.0 g; 14.08 mmol) was dissolved in acetone (70 mL) by stirring at room temperature. K_2_CO_3_ (6 eq. (11.66) g, 84.48 mmol) and ethyl bromide (5 eq., 5.2 mL, 70.4 mmol) was added. The reaction was stirred overnight. Then, the solvent was removed under reduced pressure and 70 mL water was added. The aqueous phase was extracted with ethyl acetate (3 × 60 mL) and the combined organic phases were dried over anhydrous Na_2_SO_4_ and concentrated under reduced pressure to leave a solid which was purified by trituration in hot *n*-Hexane. The compound 4-ethoxy-2-(trifluoromethyl)quinoline (compound **3** in Fig. 1) was a pale white solid with 55% yield. m. p.: 90–92 °C. ^1^H NMR (MeOD, 400 MHz) δ: 8.29 (1H, d, *J* = 8.5 Hz), 8.07 (1H, d, *J* = 8.5 Hz), 7.84 (1H, t, *J* = 8.5 Hz), 7.67 (1H, t, *J* = 8.5 Hz), 7.25 (1H, s), 4.42 (2H, q, O-CH_2_CH_3_, *J* = 4 Hz), 1.60 (t, 3H, CH_3_, *J* = 4 Hz). ^13^C NMR (MeOD, 125 MHz) δ: 165.08, 150.23 (CF_3_, *J* = 34 Hz), 149.22, 132.40, 129.82, 128.84, 123.15, 123.02, 121.54, 97.96, 66.55, 14.65. IR *v* cm^−1^: 1340 (C-O-C). Theoretical mass calculated for [C12H10F3NO + H]: 242.0793; Found: 228.0785.

#### 2.11.4 2-((2-(trifluoromethyl)quinolin-4-yl)amino)ethanol (PAMMLR-01-102-2)

A mixture of compound **3** (500 mg, 2.371 mmol) and 5 mL of ethanolamine was heated to 130 °C under stirring for 4 h when TLC analyses indicated total consumption of the starting material. Water (15 mL) was added to the reaction mixture and it was extracted with ethyl acetate (3 × 25 mL). The organic phase was dried and evaporated under reduced pressure to yield an oil which was submitted at chromatographic purification on silica gel (SiO_2_, 70–230 mesh, MeOH/CHCl_3_ 10%). The product 2-((2-(trifluoromethyl)quinolin-4-yl)amino)ethanol (compound **4** in Fig. 1) was obtained at 62% according to (Halby et al., 2017). m. p.: 173–175 °C.

#### 2.11.5 N-(2-chloroethyl)-2-(trifluoromethyl)quinolin-4-amine (PASALR-01-144)

0.3 mL of SOCl_2_ was added to a solution of compound **4** (200 mg; 0.78 mmol) in 10 mL of CH_2_Cl_2_. After 2 h of reaction at reflux, TLC indicated a total consumption of the starting material. 10% NaOH solution (20 mL) was slowly added and extracted with ethyl acetate (3 × 20 mL). The organic phase was dried with Na_2_SO_4_ and evaporated for yield a residue which was submitted to chromatographic purification on silica gel (SiO_2_, 70–230 mesh, AcOEt/*n*Hex 20%). The product *N*-(2-chloroethyl)-2-(trifluoromethyl)quinolin-4-amine (compound **5** in Fig. 1) was obtained in 75% yield according to (Halby et al., 2017). m. p.: 78–80 °C.

### 2.12 *In vitro* testing of mefloquine derivatives against *E. multilocularis* metacestodes

*In vitro* culture of *E. multilocularis* (isolate H95) metacestodes in co-culture with Reuber rat hepatoma cells was performed as described previously (Stadelmann et al., 2010). All compounds were prepared as 20 mM stocks in DMSO. The metacestode vesicle damage was assessed by phosphoglucose isomerase (PGI) assay as described previously (Stadelmann et al., 2010). In short, *in vitro* cultured metacestode vesicles of approximately 4 mm in size were extensively washed in PBS and taken up in an equal volume of DMEM without phenol red (Bioswisstec, Schaffhausen, Switzerland), including penicillin/streptomycin (100 U/mL, Thermo Fisher Scientific, Zug, Switzerland). Parasites were distributed into a 48 well-plate at 1 mL per well and mefloquine or derivatives were added to a final concentration of 10, 20, 30, and 40 μM. 0.1% Tx-100 served as a positive control, DMSO only as a solvent control. Samples were prepared in triplicates. PGI-assays were carried out after 5 and 12 days (Stadelmann et al., 2010). Active mefloquine derivatives were further tested at concentrations ranging from 40 to 1.25 μM in a 1:2 serial dilution and parasite damage by PGI-assay was assessed as described previously (Stadelmann et al., 2010). Calculations were performed in Microsoft Excel (2010), and final figures were prepared in Adobe Illustrator 2015.1.0.

## 3 Results

### 3.1 Mefloquine treatment is efficacious in mice orally infected with *E. multilocularis* eggs

**Fig. 2:**
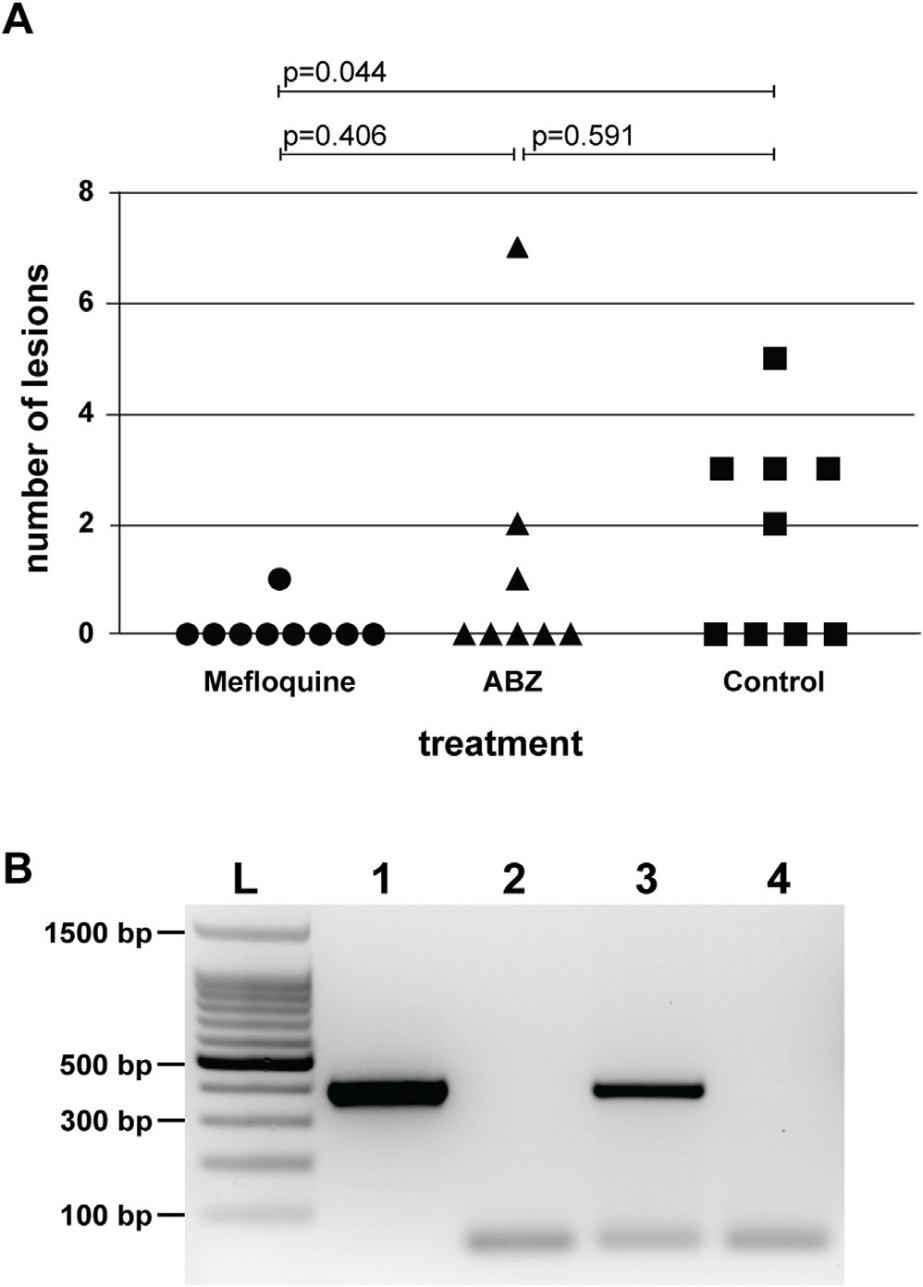
**Mefloquine treatment of *Echinococcus multilocularis* egg-infected mice.** BALB/c mice, orally infected with *E. multilocularis* eggs, were treated by either mefloquine (100 mg/kg twice per week, n = 9), ABZ (200 mg/kg, 5 times per week, n = 8) or control-treated (placebo, n = 9). After 12 weeks of treatment, parasite lesion numbers in the liver were assessed microscopically (A) and presence or absence of lesions in whole liver extracts was confirmed by PCR (B, see also Supplementary Table 1). A representative agarose gel is shown in (B) with 1, positive control; 2, negative control; 3, extract from infected mouse; 4, extract from non-infected mouse; L, 100 bp ladder.

Upon isolation of *E. multilocularis* eggs from fox intestines, sodium hypochlorite resistance test showed that egg maturity was 23 %. All mice received a dose of approximately 200 eggs. Four weeks p. i., the 26 egg-infected mice (groups I, II, and III), as well as the non-infected mice (group IV) underwent treatment for 12 weeks. No adverse effects were observed in any of the animals. Thereafter, all animals were euthanized. The number of liver lesions was assessed by stereo microscopical examination in a squeeze chamber (Fig. 2A), histological examination of each left lateral liver lobe was carried out by a pathologist, and infection of the liver tissue was confirmed by PCR (Fig. 2B, Supplementary Table 1). The blinded stereo microscopical examination of control group samples (group III, Fig. 2A) revealed the presence of *E. multilocularis* lesions in 5 of 9 mice. In ABZ-treated mice (group II), lesions were observed in 3 out of 8 mice, which was not significantly different from the placebo group (p = 0.591). In the mefloquine-treated group (group I), only 1 out of 9 mice exhibited a parasite lesion in the liver, and compared to the placebo group, the difference was statistically significant (p = 0.044) (Fig. 2A). However, compared to the ABZ-treated mice, mefloquine treatment did not lead to a significant improvement (p = 0.406). Histopathological examination of liver sections identified two animals (one each in the mefloquine treatment group (I) and the control group (III)) with *E. multilocularis* metacestodes and associated inflammation. Another two animals from the ABZ group had inflammatory lesions that could originate from parasite infection, but metacestode tissue was not clearly discernible. No further histopathological changes were detected in the liver tissue of other animals. PCR of whole livers confirmed the presence/absence of *E. multilocularis* lesions in those tissue samples previously identified by stereo microscopy (Fig. 2B, Supplementary Table 1).

### 3.2 Mefloquine plasma concentrations of infected and control-mice are similar

**Fig. 3:**
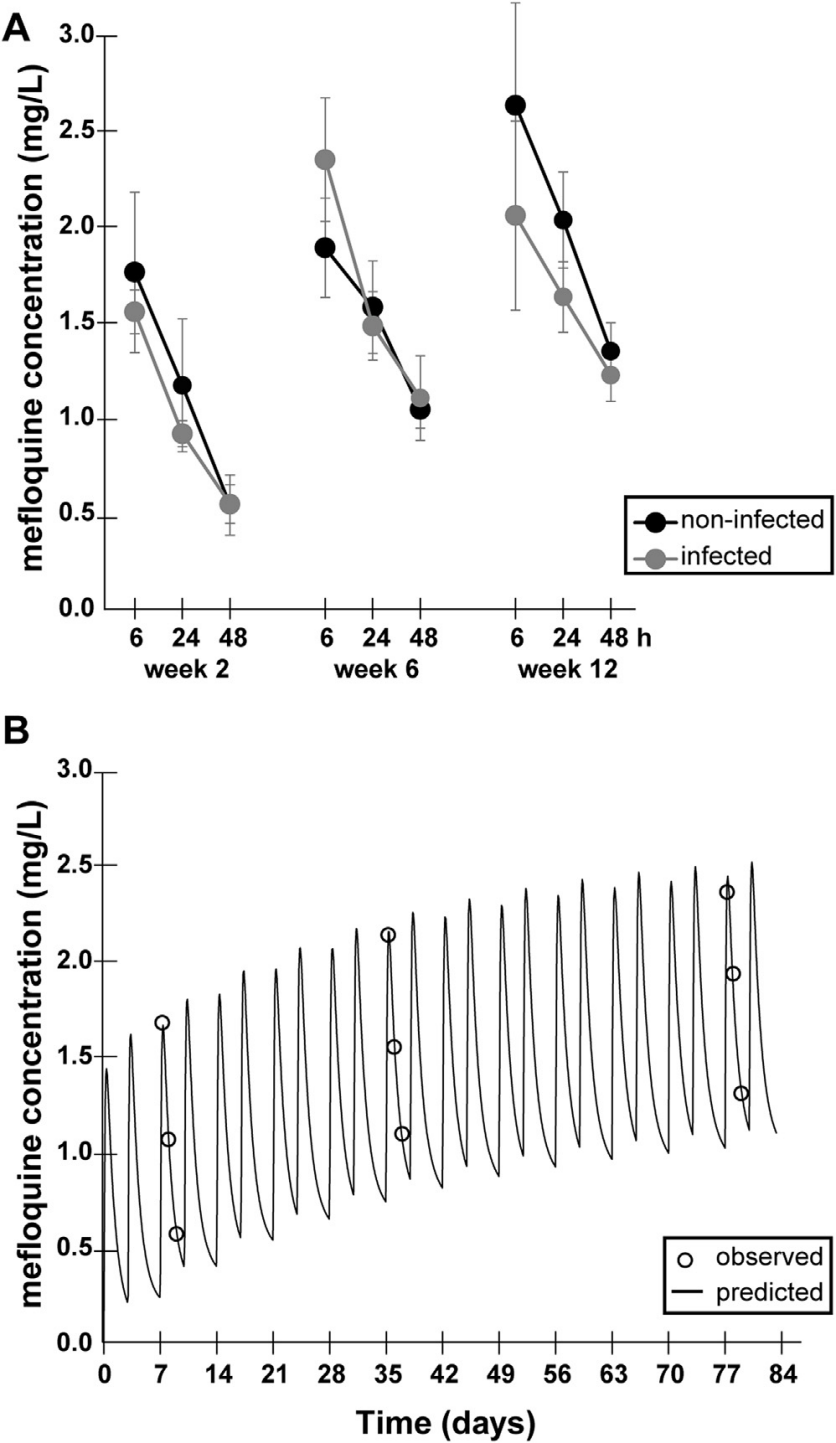
**Mefloquine plasma concentrations in BALB/c mice.** (A) Animals were treated with 100 mg/kg mefloquine per os twice per week. Plasma concentrations as assessed by HPLC are given for weeks 2, 6 and 12 of treatment. At these intervals, plasma concentrations were measured 6, 24 and 48 h after dosing (n = 3 for each time point). (B) Modeling of mefloquine concentrations as measured in (A) based on a standard two-compartment pharmacokinetic model with first-order adsorption. Predicted values are shown as solid line. Empty circles show observed mefloquine concentrations.

**Supplementary Fig. 1:**
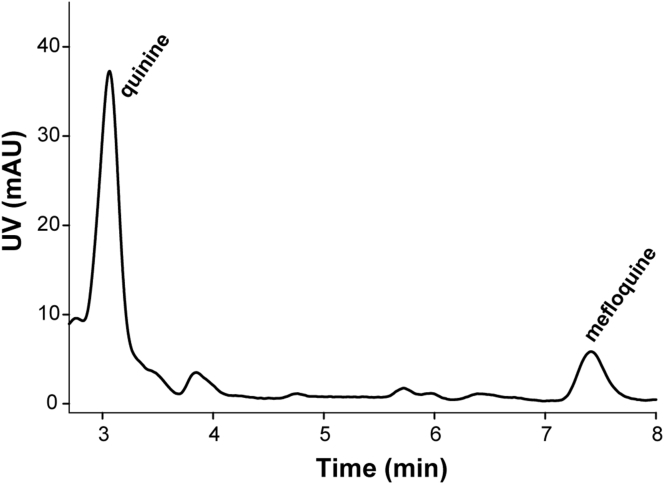
**Representative HPLC chromatogram with peaks of mefloquine and the internal standard quinine.** Chromatogram obtained with extracts from serum samples of mice treated with mefloquine and the internal standard quinidine.

During the 12 weeks mefloquine-treatment, mefloquine plasma concentrations were periodically assessed by HPLC in *E. multilocularis*-infected and corresponding non-infected animals at weeks 2, 6, and 12 of treatment. A representative HPLC chromatogram is shown in Supplementary Fig. 1. As depicted in Fig. 3A, no difference in mefloquine plasma concentrations was observed between non-infected and infected animals. There was a gradual decrease of mefloquine concentrations after dosing, with peak levels at 6 h and lowest levels measured after 48 h (Fig. 3A). At 6 h post-dosage, mefloquine concentrations reached an average (±SD) between 1.58 (±0.11) and 2.65 (±0.53) mg/L, whereas after 24 h they dropped to 0.94 (±0.07) to 2.05 (±0.25) mg/L, and after 48 h to 0.57 (±0.16) to 1.37 (±0.15) mg/L. With increasing treatment-time, mefloquine concentrations accumulated slightly (Fig. 3A).

The limited number of blood samples that could be drawn from a single mouse did not allow for individual pharmacokinetic calculations. However, to get a more comprehensive picture on the evolution of mefloquine concentrations, a pharmacokinetic analysis using average concentrations was performed (Fig. 3B). Parameter estimates were k_a_ = 0.3 h^−1^, CL/F = 0.014 L/h, CL_d_/F = 0.027 L/h, V_1_/F = 1.04 L, V_2_/F = 7.39 L. The terminal half-life of mefloquine was calculated as 580 h. After 12 weeks of treatment, the predicted steady-state trough level (C_min_ 1.15 mg/L) was reached to 91.4% and the predicted steady-state peak level (C_max_ 2.63 mg/L) was reached to 96.6%.

### 3.3 *In vitro* activity of mefloquine derivatives against *E. multilocularis* metacestodes

**Fig. 4:**
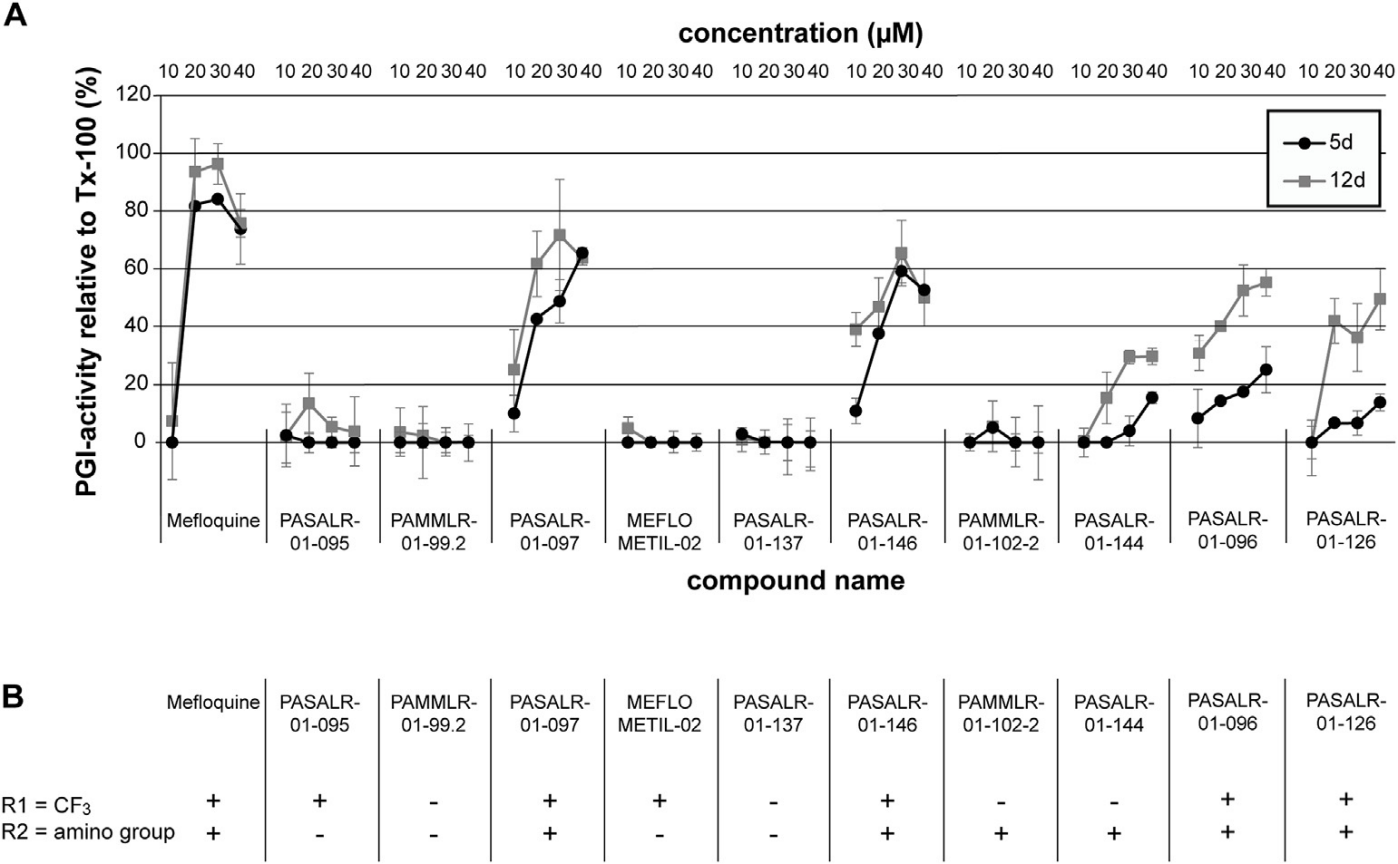
**Activity of mefloquine and ten derivatives against *E. multilocularis* metacestodes *in vitro*.** (A) Mefloquine and ten derivatives were assessed by PGI-assay for their *in vitro* activity against *E. multilocularis* metacestodes. Assessments of parasite-damage were performed after 5 and 12 days of drug-incubations at 10, 20, 30, and 40 μM in biological triplicates. Lower concentrations are not shown, as below 10 μM no activity was observed for any of the compounds listed. (B) Structural analysis of mefloquine and ten derivatives concerning presence (+) or absence (−) of a trifluoromethyl group (CF_3_) at position 8 of the quinoline structure (R1) and an amino-group containing residue at position 4 of the quinoline structure (R2).

Currently, there is no information available on which structural entities of mefloquine are important for the observed effects against *E. multilocularis*. The *in vitro* activities of 10 structural mefloquine derivatives against *E. multilocularis* metacestodes were assessed by PGI-assay. As shown in Fig. 4A, mefloquine was the most potent drug at concentrations above 20 μM, as it induced the strongest PGI-release after 5 and 12 days of treatment. Five derivatives (PASALR-01-097, PASALR-01-146, PASALR-01-144, PASALR-01-096, and PASALR-01-126) also exhibited *in vitro* activity by PGI-assay. With the exception of PASALR-01-144, which was the least active of these compounds, all other active derivatives contain a trifluoromethyl-group in the R1 residue (position 8 of the quinoline, see Figs. 1 and 4B). In addition, the R2 residue (2-piperidylmethanol substitution, see Figs. 1 and 4B) of the above-mentioned active compounds contains at least one amino group in the substitution. For none of the tested compounds did an extended incubation period of 12 days lead to much higher anti-parasitic activity, except for PASALR-01-096. At concentrations lower than 10 μM, none of the active compounds exhibited any activity against *in vitro* cultured *E. multilocularis* metacestodes.

## 4 Discussion

Over the past years, the anti-malarial drug mefloquine has been repurposed against a variety of infectious agents (Kunin and Ellis, 2000; Keiser and Utzinger, 2012; Rodrigues-Junior et al., 2016; Balasubramanian et al., 2017), and *in vivo* efficacy of mefloquine against secondary AE, induced by intraperitoneal injection of *E. multilocularis* metacestodes into mice, has been well documented (Küster et al., 2011, 2015; Gorgas et al., 2017). In this study, we have assessed the efficacy of mefloquine treatment against primary AE, caused by oral infection with *E. multilocularis* eggs, where the site of infection reflects the situation in humans. Mice were treated bi-weekly by oral gavage of 100 mg/kg mefloquine. This dose had previously been determined to be the optimal dosage against secondary murine AE in terms of achieving efficacy versus preventing adverse side effects (Küster et al., 2015). However, in those studies the plasma levels achieved by this treatment in infected and non-infected BALB/c mice were not analyzed. We here provide corresponding information and present measurements of plasma levels in weeks 2, 6, and 12 of treatment, with plasma samples obtained at 6, 24, and 48 h post-drug application. Peak-levels were expected to occur around 6–8 h after dosage based on previous studies in rodents (Ingram et al., 2013; McCarthy et al., 2016). As expected, a slight accumulation of mefloquine plasma over time was observed. Overall, mefloquine plasma levels were similar in egg-infected versus non-infected mice. This contrasts with *Schistosoma*-infected mice, where mefloquine concentrations and half-lives differed when compared to healthy control mice (Ingram et al., 2013).

In a pharmacokinetic model based on the observed mefloquine-levels, steady-state levels were predicted to be 1.15 mg/L for C_min_ and 2.63 mg/L for C_max_, and steady-state was reached to 91.4% and 96.6%, respectively, after 12 weeks of treatment, suggesting that concentrations below the predicted steady-state concentrations might be effective. In humans, the average steady-state levels after 13 weeks of a prophylactic weekly mefloquine-dosage of 250 mg mefloquine was C_max_ 1.74 (±0.34) mg/L and C_min_ 1.14 (±0.34) mg/L in one study (Pennie et al., 1993) and C_max_ 1.68 (±0.24) mg/L and C_min_ 1.12 (±0.29) mg/L in a different study (Gimenez et al., 1994). Another publication, which covered a treatment period of 21 weeks, reported steady-state levels of 0.56–1.25 mg/L (Mimica et al., 1983). Overall, the expected mefloquine concentrations in humans receiving a prophylactic weekly dosage of 250 mg mefloquine range between 0.5 and 1.7 mg/L and are thus similar, but slightly lower, to concentrations reached in mice in this study. The estimated half-life of mefloquine in mice was 580 h in this study, which corresponds closely to half-lives described for mefloquine in humans after weekly dosage for 13 weeks (422 ± 9 h (Pennie et al., 1993), or 421 ± 157 h, (Gimenez et al., 1994).

The major drawback of mefloquine are the described adverse side-effects, in particular neuropsychiatric syndrome (OR = 3.92), which includes confusion and disorientation (23.2%), dementia and amnesia (7.2%), and seizures (7.8%), as well as prodromal symptoms such as anxiety (11.3%), depression (17.4%), sleep disturbance (23.3%), and other neurological symptoms (Nevin and Leoutsakos, 2017). Serious side-effects were observed in 0.9% and 1% of mefloquine-medicated malaria patients when compared to treatment with doxycycline or atovaquone-proguanil respectively, and more detailed information on the frequency of side-effects is given in a recent Cochrane review (Tickell-Painter et al., 2017). Various biological pathways have been suggested to be involved in these neuropsychological side-effects (Gamo et al., 2010). One of them is post-hepatic syndrome, which leads to release of toxic levels of retinoids into the body, and thereby toxic neurological symptoms (Mawson, 2013). Adverse effects were also described to occur with co-medications that interfere with metabolism in the liver, as well as alcohol (Croft and Herxheimer, 2002). For these reasons, mefloquine-prophylaxis for travelers to malaria-endemic countries is not recommended for patients with a previous history of psychological disorders or alcohol abuse (Tickell-Painter et al., 2017). The advantages of mefloquine, which is still clinically applied, are the activity against chloroquine-resistant malaria, the long half-life resulting in better patient-compliance, as well as safety of use in pregnancy (Dassonville-Klimpt et al., 2011).

In mice treated with mefloquine, only one mouse out of nine had one single *E. multilocularis* lesion whereas in mice without treatment five out of nine had multiple lesions. The egg-infection model of murine AE is not as well developed as the secondary murine model of AE, and up to date assessments of parasite burden at the endpoint have relied solely on morphological observation of parasite lesions in squeezed livers by stereo microscopy. In this study, we have applied an additional and more objective assessment, by using whole-liver PCR based on the method described by Trachsel and colleagues (Trachsel et al., 2007). PCR detected parasite DNA in whole liver extracts only in those samples that were identified to contain *E. multilocularis* lesions by microscopy, thus validating the microscopy results. For the future, this method could even be expanded for a quantitative assessment of the parasite burden. Serology could be applied as an alternative method to confirm successful infection. However, as of to date, the Em2-based serology classically applied for human patients exhibited varying sensitivity in egg-infected mice (own observations). This is as also the case in dogs with AE, where sensitivity of Em2-serology ranges between 0.52 and 0.92 (Frey et al., 2017). Thus, for the murine model of primary infection with *E. multilocularis*, a better diagnostic antigen awaits to be defined.

To date, little is known regarding the mode of action of mefloquine against *E. multilocularis*. As the parasite does not rely on blood consumption, the accepted mode of action involving accumulation of toxic heme can be excluded. A deeper understanding of the structural entities that cause the profound anti-echinococcal activity, as well as of the molecular drug target(s), is needed to improve the efficacy and safety-profile of mefloquine. *In vitro* efficacy studies on 10 mefloquine-derivatives against *E. multilocularis* metacestodes showed that the trifluoromethyl group of mefloquine at the R1 position (position 8 of the quinoline structure) seems to be essential, as it is described against *Plasmodium* spp. (Dassonville-Klimpt et al., 2011). The other trifluoromethyl group (on position 2 of the quinoline structure) is also known to be essential for the potent activity against malaria (Dassonville-Klimpt et al., 2011), but this residue was not further assessed in the present study. Binding of metal ions to the trifluoromethyl groups could contribute to the mechanism of action, since the iron-binding protein ferritin was shown to bind to mefloquine *in E. multilocularis* (Küster et al., 2015). Furthermore, mefloquine inhibits hemozoin formation from the heme of the metalloprotein haemoglobin in *Plasmodium* spp. (Egan et al., 1994), as well as the magnesium-containing enolase of *Schistosoma mansoni* (Manneck et al., 2012). Upon substitution of the 2-piperidylmethanol group at the R2 position, the derivative was only active when an amino group was present. A similar observation was made by Barbosa-Lima et al. who tested 2,8-bis(trifluoromethyl)quinoline analogs against the Zika virus (Barbosa-Lima et al., 2017). For anti-malarial activity, the amino group in R2 is known to be essential, as is the hydroxyl group (Dassonville-Klimpt et al., 2011). According to our observations, the hydroxyl group does not play an essential role for anti-echinococcal activity. Within the limited number of derivatives tested here, we could show that a more electron-withdrawing substituent on the beta-position of the amine in the R2 resulted in higher activity against *E. multilocularis* metacestodes.

## 5 Conclusions

We here provide first evidence that bi-weekly mefloquine treatment in mice infected orally with *E. multilocularis* eggs at 100 mg/kg, is at least as active as 200 mg/kg ABZ applied 5 days per week. In the present study, analytical assessment of plasma levels showed that oral application of mefloquine by gavage led to plasma levels that are slightly above the described levels reached in humans taking the compound for malaria prophylaxis. Thus, there is a promising opportunity that potentially might be exploited also for the treatment of human AE. This could be of particular interest for patients that suffer from severe benzimidazole toxicity. However, due to the inherent variability of the biological material used for such infections in this model, and the limited numbers of mice that got successfully infected, further confirmatory studies need to be carried out in the future. *In vitro* structure-activity relationship studies show that the efficacy of mefloquine is highly dependent on the presence of two residues, both of which are also essential for its anti-malarial activity. Further studies will be needed to elucidate the precise mode of action of mefloquine against *E. multilocularis*.
